# Collagen-induced arthritis in common marmosets: a new nonhuman primate model for chronic arthritis

**DOI:** 10.1186/ar3172

**Published:** 2010-10-26

**Authors:** Michel PM Vierboom, Elia Breedveld, Ivanela Kondova, Bert A 't Hart

**Affiliations:** 1 Department of Immunobiology, Biomedical Primate Research Centre, Lange Kleiweg 161, 2288 GJ Rijswijk, The Netherlands; 2 Animal Science Department, Biomedical Primate Research Centre, Lange Kleiweg 161, 2288 GJ Rijswijk, The Netherlands; 3 Department of Immunology, Erasmus University Medical Center, Room Ee-828, Dr. Molewaterplein 50, 3015 GE Rotterdam, The Netherlands

## Abstract

**Introduction:**

There is an ever-increasing need for animal models to evaluate efficacy and safety of new therapeutics in the field of rheumatoid arthritis (RA). Particularly for the early preclinical evaluation of human-specific biologicals targeting the progressive phase of the disease, there is a need for relevant animal models. In response to this requirement we set out to develop a model of collagen-induced arthritis (CIA) in a small-sized nonhuman primate species (300 to 400 g at adult age); that is, the common marmoset (*Callithrix jacchus*).

**Methods:**

Twenty-two animals divided into three experiments were immunized with collagen type II (CII) of either bovine or chicken origin with different immunization strategies. The animals were analyzed for clinical manifestation of arthritis, hematology and clinical chemistry, immunological responses against CII and histopathological features of the arthritis.

**Results:**

Clinically manifest arthritis was observed in almost 100% (21 out of 22) of the animals. Fifty percent of the animals developed semi-acute CIA while the other 50% displayed a more chronic disease. Both cellular (CD3/CD4 and CD3/CD8) and humoral responses (IgM and IgG) against CII were involved in the development of the disease. Besides mild histopathological changes in bone and cartilage, severe inflammation in extraarticular tissues like periosteum and subcutaneous tissues was observed.

**Conclusions:**

This new model in marmosets more closely resembles chronic RA with respect to the chronic disease course and pathomorphological presentation than the more acute monophasic and destructive CIA model in macaques. This model can therefore fill a niche in preclinical testing of new human specific therapeutics.

## Introduction

The drug development industry continues to invest heavily in the development of new drugs against rheumatoid arthritis (RA) based on biological regulators and antagonists of proinflammatory molecules, such as monoclonal antibodies or soluble receptor molecules. The investments are based on the expectation that biological drugs will act more specifically and with less side effects than the classical broad-acting nonbiological disease-modifying and anti-inflammatory treatments. Biological molecules have their own safety problems that are not experienced with non-biological molecules, however, such as induction of neutralizing immunity or hypersensitivity reactions caused by massive cytokine release or activation of the complement system. Owing to the high species and target molecule specificity, biological drugs are often not active in lower animal models, such as mice and rats. This implies for a considerable proportion of the new biological therapeutics that the classical disease models are not a feasible option for preclinical safety and efficacy assessment, leaving nonhuman primates as the only relevant species [1].

The rhesus and cynomolgus macaque models of collagen-induced arthritis (CIA) provide useful disease models that have allowed for the efficacy evaluation of new therapeutics [2-4]. These models, however, also have a number of disadvantages. First, macaques are large-sized animals ( > 6 kg at adult age) requiring substantial quantities of test substance to achieve an effective dose. Second, the outbred nature of the model is translated into considerable clinicopathological heterogeneity between individual monkeys. As we strive to perform experiments with treatment groups comprising a small number of animals (usually five to seven animals), beneficial effects of less robust therapeutics are often overlooked. Third, the disease in macaques is often severe, short lasting and self-limiting, which limits the operational window of therapies that are administered after clinical manifestation of the arthritis.

Similar disadvantages in macaque models of another experimental autoimmune-inflammatory disorder modeled on multiple sclerosis - that is, experimental autoimmune encephalomyelitis (EAE) - have triggered the search for an alternative model that does not have these disadvantages. As reviewed elsewhere, the EAE model in common marmosets (*Callithrix jacchus*) provides a useful and often superior alternative [5-7]. The common marmoset is a small-sized Neotropical primate (±350 g at adult age) born as non-identical twins or triplets with chimeric bone marrows due to fusion of the placental bloodstreams [8]. Immune cells of the hemopoietic systems of fraternal twins distribute equally over each sibling, and cells of the T-lymphocyte and B-lymphocyte lineage are educated in the same thymic compartments. This implies that the immune systems of twins are highly comparable [9], creating the unique situation that the twin sibling can be used as the optimal control for each monkey in an experimental group. Probably the most important feature of the model is that, in the clinical and pathological presentation, EAE in marmosets more closely resembles multiple sclerosis, whereas the corresponding models in rhesus monkeys rather resemble acute neuroinflammatory diseases, such as acute disseminated encephalomyelitis [10,11].

The aim of the current study was to investigate whether the advantages of the marmoset as a model for EAE could be transferred to the CIA model. We report here that almost 100% of marmosets sensitized against commercial bovine or chicken-type collagen develop clinically manifest arthritis. About 50% of animals develop a long-lasting disease with a mainly relapsing/remitting course. In the remaining 50% of animals, a more short-lasting disease course was observed. Pathomorphological changes in arthritic joints were consistent with moderate to marked inflammation of the synovium, fibrous capsule, periosteum and subcutis, accompanied by moderate edema, reactive synoviocytes and reactive blood vessels. Loss of chondrocytes and collagen disruption and small foci of bone resorption were present. In addition, several biomarkers were evaluated that have been developed for the CIA model in the rhesus monkey [3,4,12,13] proved to be valid also in marmosets.

## Materials and methods

### Animals

Healthy male and female marmosets of adult age ( > 1.5 years) were supplied by the Institute's Animal Science Department. Individual data of the animals are presented in Table 1. Before inclusion into an experiment, each monkey received a complete health check by the veterinary staff, including assessment hematological, serological and microbiological abnormalities. During the experiments the monkeys were housed in pairs in spacious cages with cage enrichment and padded shelter provided. The daily diet during the study consisted of commercial food pellets for New World monkeys (Special Diet Services, Essex, UK) supplemented with rice, raisins, peanuts, marshmallows, biscuits, fresh fruit, grasshoppers, and maggots. Drinking water *ad libitum*, slurry enriched with vitamin D, and vegetables of the season were provided.

**Table 1:** Individual data of the monkeys

Experiment	Animal	Gender	Age (years)	**Origin of CII**^**a**^	Starting weight (g)
I	Mi119	Female	6.4	bo	344
	Mi121	Female	5.8	bo	384
	Mi125	Female	6.3	bo	364
	Mi137	Female	5.6	bo	392
II	M03136	Female	3.3	ch	325
	M03137	Female	3.3	bo	373
	M04031	Male	2.9	ch	283
	M04111	Male	2.3	ch	313
	M04121	Male	2.3	ch	299
	M05003	Female	2.0	bo	288
	M05026	Male	1.8	bo	344
	M05031	Male	1.8	ch	276
	M05032	Male	1.8	bo	316
	M05038	Male	1.8	bo	284
	M05041	Female	1.8	ch	353
	M05042	Male	1.8	bo	293
III	Mi012959	Male	3.4	ch	407
	Mi013005	Female	3.3	ch	326
	Mi013171	Male	2.9	ch	344
	Mi013385	Female	2.2	ch	362
	M06048	Female	1.8	ch	367
	M06075	Female	1.8	ch	290

^a^Collagen type II (CII) purified from bovine cartilage of the knee (bo) or of chicken origin (ch).

### Ethics

In accordance with the Dutch law on animal experimentation, all study protocols and experimental procedures were reviewed and approved by the Institute's Ethics Committee before the experiments started.

### Induction of collagen-induced arthritis

For the pilot study (Experiment I) a semi-purified batch collagen type II (CII) was prepared, extracted from bovine hyaline cartilage. For Experiments II and III, two industry-grade commercially available types of CII were used, respectively of bovine (bo-CII) or chicken (ch-CII) origin (MD Biosciences, Zürich, Switzerland). Collagen was dissolved in 0.1 M acetic acid to a final concentration of 5 mg/ml and mixed with an equal volume of complete Freund's adjuvant (CFA) (DIFCO, Detroit, MI, USA). A stable emulsion was prepared by gentle stirring of the protein/CFA emulsion for 60 minutes at room temperature. CIA was induced by injection of 0.4 ml emulsion into the dorsal skin distributed over four spots of 100 μl. The final amount of CII the animals received was 1 mg/animal. When no clinical signs of arthritis were visible at day 28, the animals were boosted with CII in incomplete Freund's adjuvant (IFA) at day 35 (Experiment II)/day 28 (Experiment III) subcutaneously in the flank.

### Clinical scoring and biomarker analysis

The presence of clinical signs was recorded by daily cage-side monitoring of behavioral changes (apathy, loss of appetite) or pain (avoidance of limb usage). Twice per week all monkeys were sedated by intramuscular injection of 0.1 ml/kg ketamine (10 mg/ml) for determination of bodyweight - an accepted surrogate disease marker for the CIA model - determination of body temperature, blood collection and inspection of the limbs for redness and/or swelling of the joints. Observations were recorded using the discomfort management schedule (Table 2).

**Table 2:** Integrated Discomfort Score

Disease score	Characteristics	Monitoring	**Maximal duration**^**a**^
0	Asymptomatic	Daily	End of experiment
	No general discomfort signs		
0.5	Fever ( > 0.5°C)	Twice per week	12 weeks
1	Apathy	Daily	10 weeks
	Less mobility but no pain	Daily	
	Loss of appetite	Daily	
2	Weight loss	Twice per week	6 weeks
	Warm extremities	Twice per week	
	Treatable pain without STS	Daily	
3	Moderate redness + STS of joints	Twice per week	4 weeks
	Normal flexibility of extremities	Twice per week	
4	Severe redness + STS of joints	Twice per week	2 weeks
	With joint stiffness		
5	Serious lethargy	Daily	18 hours
	Serious untreatable pain	Daily	18 hours
	Serious immobility of joints	Twice per week	18 hours^b^
	Body weight loss >25%	Twice per week	18 hours^b^

STS, soft tissue swelling. ^a^The discomfort time combination is used in a cumulative fashion. ^b^Can only be assessed in the sedated monkey, which cannot be done more than twice per week for ethical reasons.

### Hematology and clinical chemistry

All hematological and clinical chemistry analyses were performed at the Biomedical Primate Research Centre on a Sysmex Sf-3000 (Goffin Meyvis, Etten-Leur, The Netherlands) and a COBAS INTEGRA-400+ (Roche, Almere, The Netherlands), respectively.

### Urinalysis

Urinary excretion of the collagen crosslinks hydroxylysylpyrridinoline (HP) and lysylpyrridinoline (LP) were determined twice weekly, starting from the day of CIA induction. For that purpose, each animal's urine was collected overnight in a metabolic cage. After centrifugation, the clear supernatant was isolated and stored at -20°C. Reverse-phase high-performance liquid chromatography was used to determine HP and LP levels in hydrolyzed urine samples as described previously [14]. The levels of HP and LP were normalized to creatinine levels (nmol levels/mmol creatinine) to compensate for a possible dilution by spilled drinking water.

### Immunoassays

#### Detection of anti-collagen type II antibodies (Experiments I and II)

Serum levels of antibodies directed against bo-CII and ch-CII of IgM and IgG isotypes were detected by ELISA [15]. Serum samples (0.2 ml) were collected twice a week. Plates (96-well, F-form, microlon; Greiner bio-one, Alphen aan den Rijn, Netherlands) were coated overnight at 4°C with 100 μl of a 10 μg/ml solution of bo-CII/PBS. Plates were washed four times with PBS + 0.05 Tween-20 and subsequently blocked by a 1-hour incubation with 200 μl PBS + 1% BSA at 37°C and 5% CO_2_. Plates were washed four times, after which 100 μl diluted serum was added (CII-IgG => 1:25; IgM => 1:25) in PBS + 1% BSA and incubated overnight at 4°C. After an additional incubation for 2 hours at 37°C, 5% CO_2 _in a humidified atmosphere, plates were washed four times with PBS + 0.05 Tween-20.

Plates were subsequently incubated for 1 hour with a secondary detector antibody (AP-conjugated goat anti-human IgM_1_, 10,000 in PBS + 1% BSA or AP-conjugated goat anti-human IgG_1_, 10,000 in PBS + 1% BSA; Biosource, Camarillo, CA, USA). After washing, antibody binding was detected by adding 100 μl poly-nitrophenylphosphate diluted in Tris buffer (Sigma Chemicals, Zwijndrecht, The Netherlands). Color development was determined by absorbance at 405 nm.

#### Flow cytometry and carboxyfluorescein succinimidyl ester staining (Experiment II)

To determine the phenotype of proliferating cells, 4 × 10^6 ^viable peripheral blood mononuclear cells (PBMC), axillar lymph node mononuclear cells, inguinal lymph node mononuclear cells and spleen mononuclear cells were suspended in 1 ml PBS and incubated for 7 minutes at room temperature with carboxyfluorescein succinimidyl ester (final concentration, 1.5 μM; Fluka, Buchs, Switzerland). The labeled cells were cultured for 7 days with antigens under the standard culture conditions described above. For flow cytometric analysis we used the following commercially available labeled mAbs directed against human CD markers: anti-CD3 with PerCP or Alexa Fluor 700 label (BD Biosciences, San Jose, CA, USA), allophycocyanin-labeled anti-CD4 (DakoCytomation, Glostrup, Denmark) and biotinylated anti-CD8 (Serotec, Düsseldorf, Germany). Flow cytometric analysis was performed on a FACSort flow cytometer using FACSDiva software (BD Biosciences). First, viable cells were gated using the live/dead fixable violet viability stain (Invitrogen Life Technologies, Breda, The Netherlands). Within the viable cell gate, lymphocytes/monocytes were selected using forward and sideward scatter. Within the lymphocyte/monocyte gate, CD3^+ ^cells were selected. The CD3^+ ^population in the carboxyfluorescein succinimidyl ester experiment consisted of CD4^+ ^and CD8^+ ^cells.

#### *Ex vivo *analysis of proliferative responses (Experiment III)

The maximum blood sample that can be collected in 1 month from primates at the Biomedical Primate Research Centre should not exceed 1% of the body weight. For an average adult common marmoset weighing 350 g, this equals a maximum monthly blood sample of 3.5 ml. Hence, volumes of up to 1 ml at 4-week intervals were collected into heparinized Vacutainer tubes (Greiner bio-one). To measure the development of ch-CII-specific cellular responses with time, PBMC were isolated from heparinized venous blood using lymphocyte separation medium (ICN Biomedical, Aurora, OH, USA).

In addition, cell suspensions were prepared at necropsy from aseptically removed axillar lymph nodes, inguinal lymph nodes and spleen. PBMC, lymph node cells and spleen cells were cultured in RPMI medium (HEPES-buffered) supplemented with 10% FCS, 2 mM L-glutamax, 100 U/ml penicillin, 100 μg/ml streptomycin and 2 × 10^-5 ^M 2-Mercaptoethanol (all obtained from Life Technologies) at 37°C in humidified air containing 5% CO_2_. Cultures were tested in triplicate for the detection of proliferative responses towards ch-CII and an overlapping peptide set derived from the immunogenic cyanogen bromide fragment 11 (CB11) fragment (Table 3; CII_aa 124 to 402_). All peptides were dissolved in PBS and tested at a final concentration of 5 μM. ch-CII was dissolved in 0.1 M acetic acid (5 mg/ml), denatured at 42°C for 30 minutes and tested at a final concentration of 10 μg/ml. The proliferative response was measured as [^3^H]thymidine incorporation. After 72 hours of culture, 0.5 μCi [^3^H]thymidine was added per well, and incorporation of the radiolabel was determined after 18 hours using a matrix 9600 beta counter (Packard, Meridan, CT, USA). Results of ch-CII-specific proliferation are expressed as the mean stimulation index relative to the medium control:

**Table 3:** Overlapping peptide set derived from chicken-origin collagen type II fragment number 11 after cyanobromide digestion

Name	**Amino acid numbers**^**a**^	Amino acid sequence 1234567890123456789012345
pep 1	124 to 149	GPRGLPGERGRPGPSGAAGARGNDG
pep 2	140 to 165	GAAGARGNDGLPGPAGPPGPVGPAG
pep 3	155 to 180	GPPGPVGPAGAPGFPGAPGSKGEAG
pep 4	170 to 195	GAPGSKGEAGPTGARGPEGAQGPRG
pep 5	185 to 210	GPEGAQGPRGESGTPGSPGPAGAPG
pep 6	200 to 225	GSPGPAGAPGNPGTDGIPGAKGSAG
pep 7	215 to 240	GIPGAKGSAGAPGIAGAPGFPGPRG
pep 8	230 to 255	GAPGFPGPRGPPGPQGATGPLGPKG
pep 9	245 to 270	GATGPLGPKGQTGEPGIAGFKGEQG
**pep 10**^a^	**251 to 276**	GPKGQTGEPGIAGF**K**GEQGPKGETG
pep 11	260 to 285	GIAGFKGEQGPKGETGPAGPQGAPG
pep 12	275 to 300	GPAGPQGAPGPAGEEGKRGARGEPG
pep 13	290 to 315	GKRGARGEPGAAGPVGPPGERGAPG
pep 14	305 to 330	GPPGERGAPGNRGFPGQDGLAGPKG
pep 15	320 to 345	GQDGLAGPKGAPGERGPAGLAGPKG
pep 16	335 to 360	GPAGLAGPKGATGDPGRPGEPGLPG
pep 17	350 to 375	GRPGEPGLPGARGLTGRPGDAGPQG
pep 18	365 to 380	GRPGDAGPQGKVGPTGAPGEDGRPG
pep 19	370 to 395	GAPGEDGRPGPPGPQGARGQPGVMG
pep 20	374 to 400	EDGRPGPPGPQGARGQPGVMGFPGP

Overlapping peptide set derived from chicken-origin collagen type II (ch-CII) fragment number 11 after cyanobromide digestion and separation on high-performance liquid chromatography (CB11; ch-CII_aa 124 to 402_). ^a^ch-CII amino acid 124 = aa_1_. ^b^This peptide contains the immunodominant glycopeptide [29].

Stimulation index=cpm experimental sample/cpm medium control.

where cpm is counts per minute. SI >2.0 was considered relevant.

## Results

### Incidence of collagen-induced arthritis

**Figure 1:**
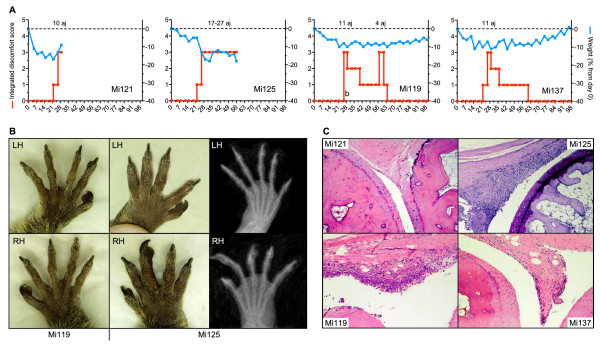
**Collagen-induced arthritis induced with bovine collagen type II purified from bovine cartilage of the knee**. **(a) **Clinical signs of active arthritis, such as soft-tissue swelling and redness of affected joints, were scored twice a week using the semiquantitative scale Integrated Discomfort Score (see Table 2; red line). Number of affected joints (aj) involved in the arthritis is indicated above the dashed line. The monkeys were weighed twice a week, body weight serving as a surrogate disease marker. Body weight changes over time are expressed as the percentage of weight relative to day 0 (blue line). **(b) **A clear difference in swelling was observed between Mi119 (no swelling) and the prominent swelling in joints of Mi125. Structural changes could not, however, be visualized in corresponding radiograph of these joints. LH, left hand; RH, right hand. **(c) **Histology was performed on collagen-induce arthritis-affected proximal interphalangeal joints. Hyperplasia of the synovium was observed in joints that were clinically affected (Mi125). Signs of synovitis were also observed, however, in clinically nonaffected joints (Mi119; lymphocytic infiltrate).

Three independent experiments have been performed, involving a total of 22 marmosets. In the first experiment, four marmosets were immunized according to the standard protocol that was developed for CIA induction in genetically susceptible rhesus monkeys; that is, a single immunization with semi-purified bo-CII in CFA [14,16]. All four monkeys displayed signs of CIA; that is, painful joints, expressed by relief of pressure from the hind legs and reluctance to manually take an offered reward (marshmallow), and a marked loss of body weight (Figure 1a). Sustained clinical arthritis, however, was observed in only two monkeys (Mi121 with 10 affected joints at day 29, and Mi125 with >20 affected joints at necropsy; Figure 1a). The two remaining animals displayed minimal clinical signs of arthritis transiently and received a subcutaneous booster immunization with bo-CII in IFA at day 114. This did not aggravate joint swelling but did boost the induction CII-specific IgM and IgG in Mi137. Figure 1b shows the prominent joint swelling in the extremities of the most severe arthritic case, Mi125. The X-ray scans depicted in the same figure, however, do not show prominent bone deformations in the affected hands. Histologically, varying degrees of synovitis were observed in the joints of two out of the four monkeys (except Mi121 - which was found dead in the cage on day 30 - and Mi137). Mi125 showed prominent signs of synovitis (Figure 1c). Furthermore, histologically Mi119 displayed signs of synovitis in clinically nonaffected joints. Erosion of the cartilage surface was observed to start to develop in both Mi125 and Mi119. It was concluded from this experiment that the CIA induction protocol developed for the rhesus monkey was suboptimal for the marmoset, probably because marmosets were less susceptible to the disease.

**Figure 2:**
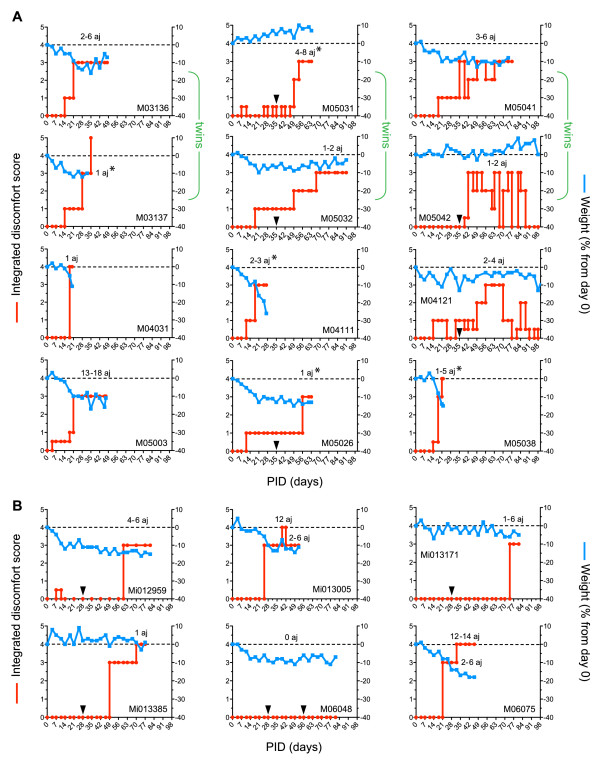
**Development of collagen-induced arthritis is independent of the source of collagen type II**. **(a) **Body weight changes over time are expressed as the percentage of weight relative to day 0 (blue line), and clinical signs were analyzed twice a week and summarized in an Integrated Discomfort Score (red line). The number of affected joints (aj) involved in the arthritis is indicated above the dashed line. Group I was immunized with chicken-origin collagen type II (ch-CII): M03136, M05031*, M05041, M04031*, M04111* and M04121. Group II was immunized with bovine-origin collagen type II: M03137*, M05032, M05042, M05003, M05026* and M05038*. Animals were boosted on day 35 if they did not develop clinical arthritis at day 28 (black triangles). The upper two lines of graphs are twin siblings (green accolade). *Sacrificed just after the onset of clinical disease. **(b) **ch-CII reproducibly induces collagen-induced arthritis (CIA) with high incidence but variable disease course. PID, post induction day.

In a second experiment, industry-grade commercial batches of CII preparations of bovine and chicken origin were tested. The immunization protocol comprised inoculation with CII in CFA (intracutaneously) followed by booster immunization with CII in IFA (subcutaneously). From each immunization group of six animals, three animals were sacrificed at the peak of the disease for histopathological examination just after the onset of clinical disease. The remaining three animals per immunization group were allowed to develop full clinical arthritis. The animals were selected alternately to be sacrificed early or late in the disease course. Clinical signs of CIA were observed in all 12 cases (Integrated Discomfort Score ≥3), but no major differences between both collagen batches were observed (Figure 2a). A relapsing/remitting disease course with only moderate weight loss was observed in several cases, whereas in other monkeys a rather acute progressive course with serious weight loss was observed. This same clinical heterogeneity is observed in marmoset models of EAE [17]. This is probably a reflection of the genetically determined responsiveness to the collagen immunization, as can be seen in the twin pairs depicted in the top of Figure 2a.

To establish reproducibility of the induction protocol, ch-CII was selected for the third experiment (*n *= 6) - the most commonly used autoantigen for CIA induction in rodents. The images in Figure 2b confirm the high incidence of CIA in this species (five out of six animals), as well as the variable disease course. The one case in which clinical signs were not observed during the 100-day observation period did display weight loss, suggestive of subclinical disease.

In conclusion, sensitization of marmosets against CII by a first immunization with collagen in CFA followed by booster immunizations with collagen in IFA reproducibly induces clinical arthritis in marmoset monkeys.

### Clinical and pathological presentation

**Figure 3:**
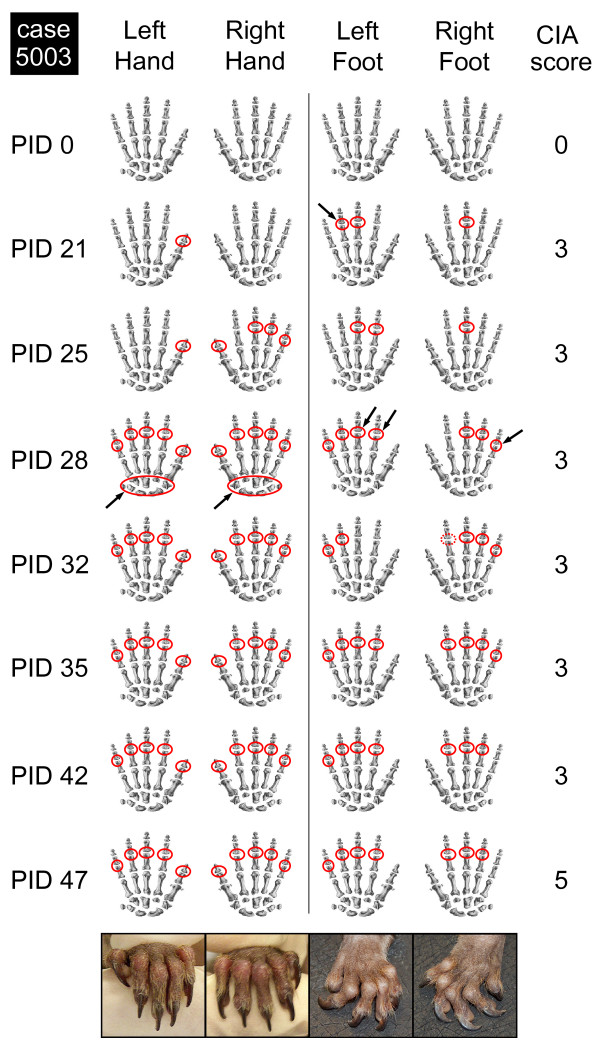
**Variable expression of clinical arthritis at the level of a single joint**. The development of clinical arthritis (mild soft tissues swelling = clinical score 3) post induction day (PID) at the levels of single joints (proximal and distal interphalangeal joints and metacarpal region) is indicated in red. If the swelling was inconclusive, this is indicated with a dashed red line (see right foot and index finger, PID 32). Black arrows point to the variable swelling of the indicated joints. CIA, collagen-induced arthritis.

The alternation of overt arthritis with episodes of complete remission that was observed in several cases could also be observed at the level of a single joint. As an example, data from M05003, which was immunized with bo-CII in Experiment II, are shown in Figure 3. Swollen joints at a given time point are indicated with red circles, while the arrows indicate joints that were found only transiently affected. The pictures at the bottom of Figure 3 show the severe swelling of interphalangeal joints and the severe deformation of the extremities at the end stage of the observation period.

**Figure 4:**
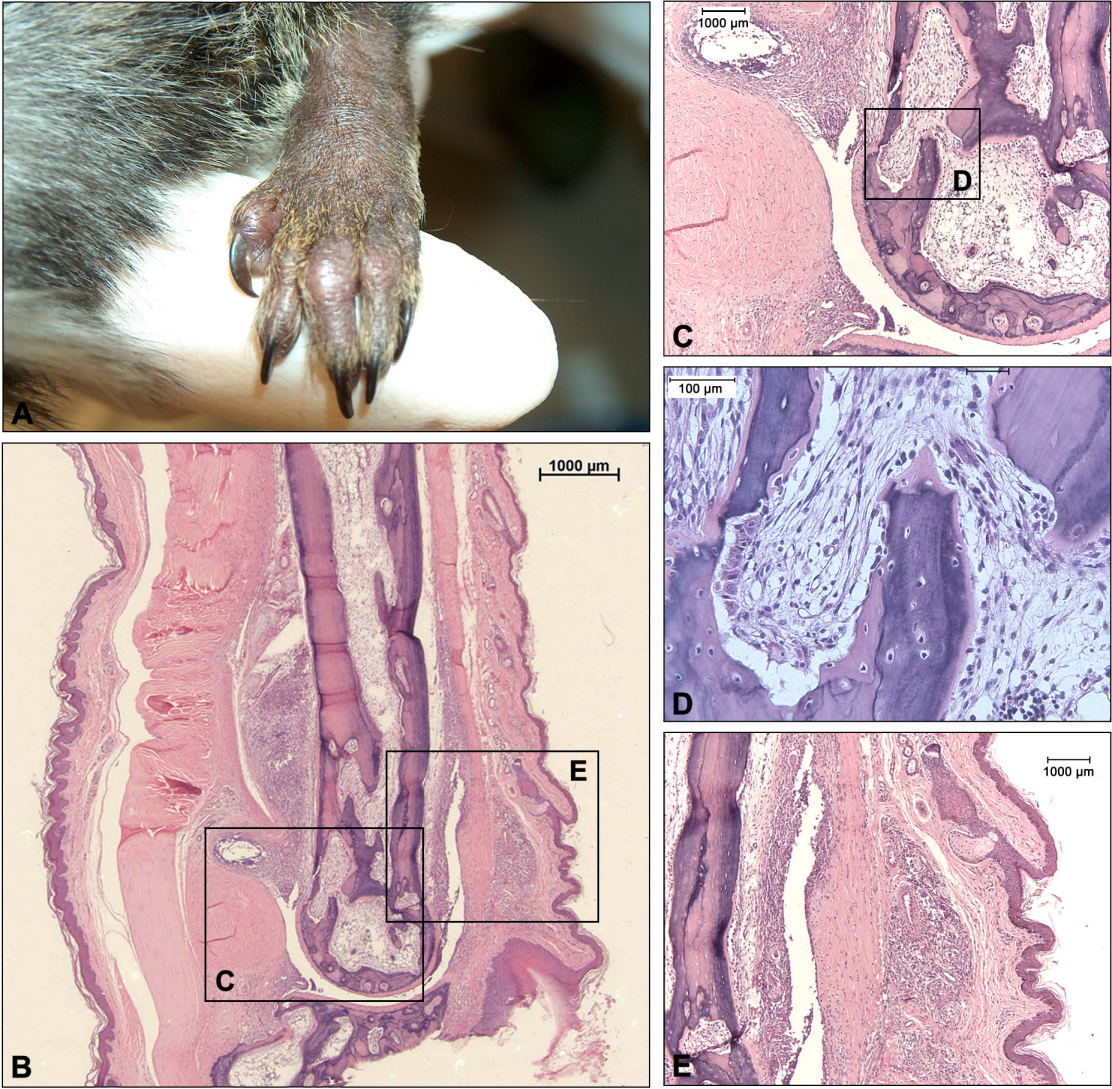
**Histology of affected joints of animals with early-onset arthritis induced with chicken-origin collagen type II**. Histology of the affected joint of a representative animal with early-onset arthritis (M03136) induced with chicken-origin collagen type II. **(a) **The swollen joint, subsequently analyzed for histopathology in panels (b) to (e). **(b) **Overview of the affected joint. **(c) **Focus on the phenomena observed in (b) displaying prominent extraarticular infiltrates and highly active bone marrow, **(d) **resulting in erosion of the bone and disruption of the cartilage. **(e) **Manifestation of subcutaneous inflammation and infiltrates at the periosteum.

Histological features of a representative animal with early onset arthritis (M03136) induced with ch-CII are depicted in Figure 4. A cross-section of a severely swollen joint (Figure 4a) was stained with H & E (Figure 4b). Figure 4c shows marked extraarticular inflammation (synovium and fibrous capsule) and loss of chondrocytes and collagen disruption. Figure 4d zooms in on an area where marked bone resorption takes place. Figure 4e shows the severe extraarticular inflammation of the periosteum and subcutis. Interestingly animals that showed marked inflammation at either the periosteum or the subcutis were mainly found in the early responders to induction (6/12 animals), which were also associated with a marked loss of body weight. Inversely, animals that showed a more chronic development of the disease (5/12 animals) displayed no such extraarticular phenomena.

### Disease biomarkers

#### Inflammation

**Figure 5:**
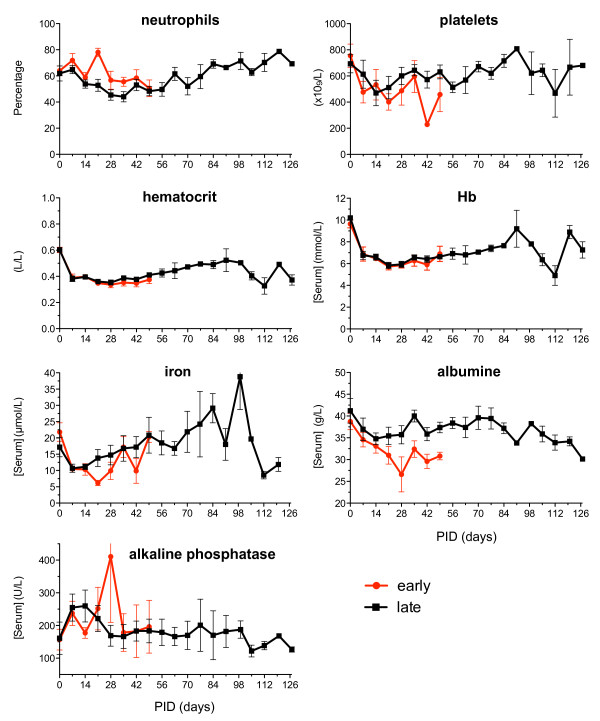
**Differences in hematological parameters between early-onset arthritis and chronic development**. No major differences in hematological parameters were observed in animals with early onset (red line) of disease and animals with a more chronic development (late = black line) of arthritis. Hb, hemoglobin; PID, post induction day.

C-reactive protein is a useful serum marker of the acute phase reaction in the rhesus monkey model of CIA. This marker could not be detected, however, as the detection reagents did not cross-react with C-reactive protein of marmosets. Hematological markers such as neutrophils and platelets were therefore analyzed on a weekly basis. Both populations remained constant during the observation period. Inflammation-induced anemia was observed, resulting in reduced hemoglobin and iron and reduced hematocrit values (Figure 5). The anti-inflammatory protein albumin shows a marked reduction in the acute responders while the late responders show a limited reduction, which recovers later on.

#### Joint erosion

In the rhesus monkey model of CIA, the urinary excretion rate of the major collagen crosslinks LP and HP was identified as a valid biomarker of joint erosion.

**Figure 6:**
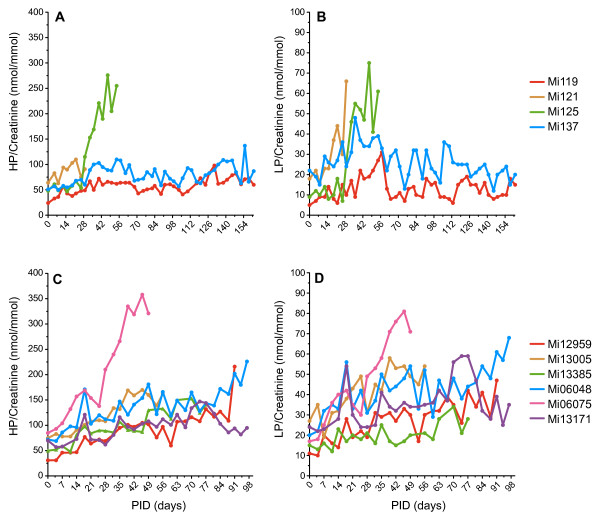
**Urine excretion rates of the collagen crosslinks lysylpyrridinoline and hydroxylysylpyrridinoline**. Urine samples were collected twice weekly and the excretion rates of the major collagen-crosslinks **(a), (c) **hydroxylysylpyrridinoline (HP) and **(b), (d) **lysylpyrridinoline (LP) were determined as described in Materials and methods. Urinary excretion rates were normalized to creatinine levels to compensate for a possible dilution factor. In Experiment I, two animals with overt clinical arthritis (Mi121, Mi125) show increased production from either (a) HP and/or (b) LP. In Experiment III, the production of (c) HP and (d) LP was less affected by the disease. Only in animals with a high number of joints involved in the disease ( > 10 affected joints) was an increased production of both HP and LP observed. PID, post induction day.

In Experiment I, a full 24-hour urinary collection was performed to measure HP and LP excretion. Animals that did not develop overt signs of arthritis (Mi119, Mi137) displayed stable HP excretion, while the one animal affected by joint swelling (Mi125) showed a marked increase in HP and LP excretion (Figure 6a, b).

In Experiment II, the time for which the animals stayed in the metabolic cage was reduced to 16 or 17 hours to limit the discomfort experienced as a consequence of isolation and separation. The amount of urine excreted by the common marmoset is far less (2 to 10 ml) then that observed with the rhesus monkey (300 to 400 ml), resulting in a large variation in measurements (data not shown).

In Experiment III, we returned to the 24-hour collection to compensate for the circadian rhythm in urine excretion observed for HP/LP excretion. In this experiment it was observed that the production of HP and LP was less affected by the disease (Figure 6). Only severely diseased animals show a marked change in the excretion of HP/LP.

### Profiles of autoreactive T cells and antibodies

The autoimmune attack on the joints in CIA models in mice, rats and rhesus monkeys is mediated by interplay of anti-CII T cells and humoral factors. In both rodent CIA models [18] as well as the CIA model in the rhesus monkey [16], a key pathogenic role of IgM autoantibodies was found. Rhesus monkeys that were genetically resistant to CIA were found incapable of producing adequate amounts of anti-collagen IgM [19]. Second, pre-sensitization of rhesus monkeys with heat-denatured CII not only abrogated the capacity to produce anti-CII IgM, but also induced resistance to CIA [20]. The relation between both autoimmune parameters and disease was therefore also tested in the novel CIA model in marmosets.

#### Antibodies

**Figure 7:**
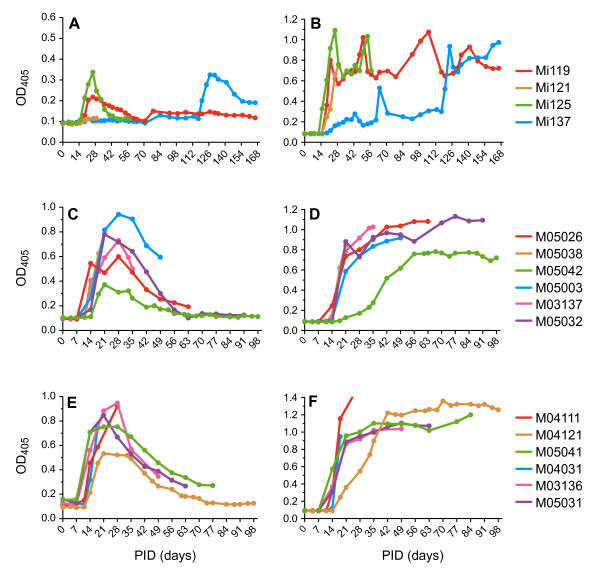
**Humoral responses play a prominent role in development of collagen-induced arthritis in the common marmoset**. In Experiment I where a semi-purified collagen type II (CII) was used, **(a) **a transient CII-specific IgM response was followed by **(b) **a sustained CII-specific IgG response. Even in animal Mi137 this phenomenon was observed after the booster at day 110. In Experiment II where commercial batches of CII were used both against **(c), (d) **bovine CII and **(e), (f) **chicken CII, a transient CII-specific IgM response ((c) and (e)) was followed by a sustained CII-specific IgG response ((d) and (f)). PID, post induction day.

In Experiment I, all animals produced CII-specific IgM and IgG (for animal Mi137 only after boosting on day 114; Figure 7a, b). For Experiment II, the induction protocol was adapted to an immunization-boost protocol resulting in reproducible transient production of CII-specific IgM (Figure 7c, e) followed by a sustained production of CII-specific IgG in all 12 animals (Figure 7d, f). Since this analysis requires substantial amounts of serum, blood was selected for use in Experiment III for a longitudinal analysis of cellular responses.

#### T cells

Two types of analysis have been performed with cells from the monkeys. A longitudinal analysis of T-cell immunity development was performed with freshly isolated PBMC (Experiment III) at 28-day interval and a biodistribution analysis of CII-responsive T cells at necropsy. Moreover, we performed a fine specificity analysis of the T-cell response against CII as well as CD4/CD8 phenotyping of the CII-responsive T cells (Experiment II). Owing to limitations in the available venous blood volume that can be collected per month during the course of a study, this assay was performed only at necropsy when sufficient cells could be obtained.

**Figure 8:**
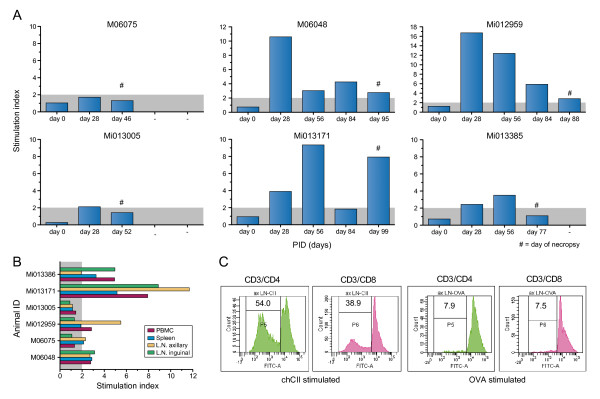
**T-cell proliferation responses**. **(a) **From the six animals in Experiment III, a longitudinal analysis of collagen type II (CII)-specific T-cell responses in freshly isolated peripheral blood mononuclear cells (PBMC) shows a variable response against denatured CII (animal identification at top of the graph). ^#^Day of necropsy. **(b) **At the day of necropsy, CII-specific proliferative responses were measured from the six animals in Experiment III. Cells isolated from the lymph nodes (spleen, axillar and inguinal) show better responses than cell isolated from the PBMC. **(c) **From 10 animals in Experiment II, the CII-specific responses were analyzed using dilution of the vital dye carboxyfluorescein succinimidyl ester in combination with cellular staining for the T-cell markers CD3, CD4 and CD8. It was shown for four out of the 10 animals analyzed that, especially in axillar and inguinal lymph nodes (LN), both CD3^+^/CD4^+ ^T cells and CD3^+^/CD8^+ ^T cells contribute to the CII-specific cellular responses (a representative experiment of those four animals is presented; animal M04111). Stimulation index = proliferation of experimental sample (counts per minute)/proliferation of medium control (counts per minute). ch-CII, chicken-origin collagen type II; PID, post induction day.

Figure 8a shows that T-cell responses in peripheral blood are variable and do not correlate with the course of the disease. Higher responses can be measured in spleen and lymph nodes, especially the axillary lymph nodes (Figure 8b). From the 12 animals that were used in Experiment II, 10 animals were analyzed for anti-CII T-cell proliferation. Using dilution of the vital dye carboxyfluorescein succinimidyl ester in combination with monoclonal antibody staining, the phenotype of CII-responsive cells was determined. In four animals we could show CII-specific responses especially in axillar and inguinal lymph nodes (an example is shown in Figure 8c). Both CD3^+^/CD4^+ ^T cells and CD3^+^/CD8^+ ^T cells seem to contribute to the CII-specific response. In three animals we could not demonstrate a CII-specific cellular response, and in three animals the data could not be analyzed due to technical failures.

**Figure 9:**
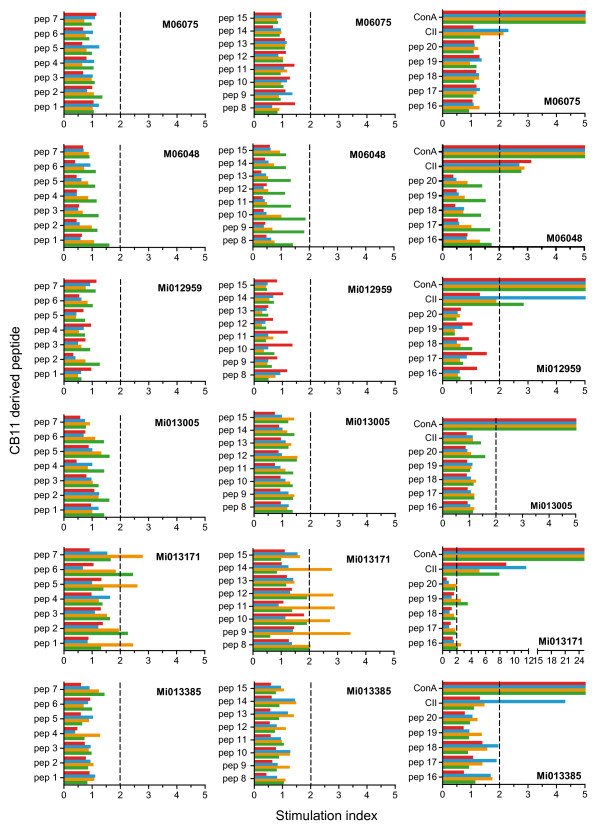
**Fine specificity of proliferating T cells**. A detailed analysis of collagen type II (CII)-specific T-cell responses against a cyanogen bromide fragment 11 (CB11)-derived overlapping peptide set (see Table 3) was performed on the day of necropsy with freshly isolated peripheral blood mononuclear cells (PBMC) (green) and cells isolated from the inguinal (red) and axillar (blue) lymph nodes and the spleen (yellow) (animal identification given in the graph). Concanavin (Con) A stimulation was included to establish functional proliferation of the different cell populations and displayed stimulation indices ranging from 25 to 160. In general, CII-specific proliferative responses are better measured in lymph nodes (spleen, axillar and inguinal) than in PBMC. Stimulation index = proliferation of experimental sample (counts per minute)/proliferation of medium control (counts per minute).

In an attempt to determine the fine specificity of the proliferating T cells, proliferation was tested against a panel of 25-mer overlapping peptides (Table 3) spanning the CB11 fragment of ch-CII (CII_aa 124 to 402_). In none of the cultures, however, could a positive response be detected (Figure 9). The epitope against which the CII-responsive T cells were directed could therefore not be determined.

## Discussion

We report the development of a new preclinically relevant model of arthritic disease in the common marmoset. The marmoset is a small-sized Neotropical primate that gains increasing popularity as a model of human diseases. The advantages compared with other frequently used nonhuman primates, such as the rhesus monkey and cynomolgus macaque, are obvious. Marmosets breed easily in captivity, giving birth to four siblings per couple per year, which reach adulthood after 1.5 years of age. The small size, 350 to 400 g at adult age, implies that much lower amounts of test substance can be used compared with the more than 10-fold larger macaques. A particularly attractive aspect is that marmosets are born as complete bone marrow chimeras, which implies a high immunological similarity. This principle can be used in therapy trials with one twin sibling as the recipient of experimental treatment and the other sibling the recipient of placebo. Moreover, the allotolerance between twin siblings allows the transfer of suspected pathogenic T-cell specificities - a standard research tool in inbred rodent disease models, but one that is precluded by the genetic diversity in outbred macaque models.

For the development of a CIA model in the marmoset we relied on our experience with rat and rhesus monkey models of CIA [12,14-16] and with the marmoset as a model of the chronic progressive immune-mediated inflammatory disorder multiple sclerosis, EAE [5,7,10,11]. We chose to examine whether a similarly valid model for chronic progressive arthritis as for multiple sclerosis could be developed in the marmoset. The only difference between EAE and CIA is the immunizing antigen, being recombinant human myelin/oligodendrocyte glycoprotein for the EAE model and type II collagen for the CIA model.

Similar to the EAE model, we observed in the CIA model an almost 100% disease incidence (only one outlier in 22 cases) and a heterogeneous clinical course. This feature probably reflects the genetic heterogeneity of this outbred species. The variety of disease patterns included acute cases with a rapid disease onset without remissions, relapsing/remitting cases with alternating episodes of inflammation and recovery, and progressive cases with slowly incrementing disease scores. Especially in the more chronic cases, inflammation of individual joints is disseminated in time and space, suggesting competition between systemically acting proinflammatory mechanisms with locally acting anti-inflammatory mechanisms. This principle was also observed in the EAE model and has helped to identify the specificity and mode of action of the core pathogenic T cells (reviewed in [11]).

Histological examination of affected joints showed the same pathological aspects as observed in the rhesus monkey CIA model - namely, strong synovial hyperplasia forming pannus tissue overgrowing and eroding the cartilage surface and degradation of subchondral bone [3,4]. Besides the clear articular pathology, extraarticular pathology could also be observed - such as inflammation of the subcutaneous tissue, inflammation of the periosteum, development of perivasculitis, presence of reactive blood vessels and angiogenesis. Together, the features discussed thus far mark a considerable improvement compared with the acute self-remitting CIA model in rhesus monkeys.

For the immune profiling we have focused on parameters that were found of pathogenic relevance in the rhesus monkey CIA model - namely, the capacity to generate IgM and IgG autoantibodies [14,20] and the presence of a T-cell proliferative response [19]. One of the few disadvantages of the marmoset model is the low amount of blood that can be collected for analysis of cellular immune reactions, which is about 3 ml per month. T-cell proliferation in PBMC was therefore only assessed once every 28 days. The results show a variable proliferative response, which displayed no obvious relation with the clinical course. The analysis at necropsy showed that the vast majority of CII-responsive T cells is located in the lymphoid organs, spleen and axillary lymph nodes in particular, which most probably explains this poor discrepancy. Only at necropsy could sufficient cells be obtained from blood and lymphoid organs for phenotyping of the CII-responsive cells and a fine specificity analysis of the anti-CII T-cell response. Interestingly, in the axillary lymph nodes that are located in the armpits, we observed not only the expected specific proliferation of CD4^+ ^T cells, but also proliferation of CD8^+ ^T cells. Although in several monkeys good proliferative responses were detected against the complete CII protein, none of the synthetic 25-mer peptides derived from the immunogenic CB11 fragment of ch-CII could stimulate proliferation.

## Conclusions

The common marmoset may fill a niche as an attractive alternative nonhuman primate species for preclinical evaluation of new biologicals in the field of arthritis. The small size of the common marmoset compared with the rhesus monkey translates into lower caging, feeding and housing costs. This advantage coupled with the lower purchase price of common marmosets compared with macaques may translate into substantial cost savings when performing equivalent studies in rhesus monkeys. Furthermore, the marmoset is easier to handle than the aggressive rhesus monkeys, which need to be sedated for every handling [21,22]. Of note is the fact that the evolutionary distance between the common marmoset and human is 33 million years while the evolutionary distance between the rhesus monkey and human is 23 million years [23]. This translates into significant homology differences between human and the common marmoset, which is demonstrated by the fact that a substantial number of ELISAs that crossreact with rhesus cytokines do not crossreact with cytokines from the common marmoset (personal observation). Evaluation of a promising new compound for RA in the common marmoset will always require establishing crossreactivity of this new compound for targets in the common marmoset. For this purpose we have stored tissue in Oxidized Regenerated Cellulose at -80°C, allowing for immunohistochemistry and isolated mononuclear cells from axillar and inguinal lymph nodes, spleen and blood in liquid nitrogen for functional assays.

The common marmoset already plays an important role in diverse areas of research such as infectious diseases [24,25], stem cell research, neural and cognitive sciences [5,10,26], reproductive biology, toxicology and drug development [27,28]. We believe in the years to come that the CIA model in the common marmoset may fill a void for drug development in the area of immune-mediated inflammatory diseases.

## Abbreviations

bo-CII: bovine-origin collagen type II; BSA: bovine serum albumin; CB11: cyanogen bromide fragment 11; CFA: complete Freund's adjuvant; ch-CII: chicken-origin collagen type II; CIA: collagen-induced arthritis; CII: collagen type II; EAE: experimental autoimmune encephalomyelitis; ELISA: enzyme-linked immunosorbent assay; FCS: fetal calf serum; H & E: hematoxylin and eosin; HP: hydroxylysylpyrridinoline; IFA: incomplete Freund's adjuvant; LP: lysylpyrridinoline; mAb: monoclonal antibody; PBMC: peripheral blood mononuclear cells; PBS: phosphate-buffered saline; RA: rheumatoid arthritis.

## Competing interests

The development of this new model was financially supported by unbiased grants from GSK and Roche.

## Authors' contributions

MPV designed and executed the total study, was responsible for the physical examination of the animals during the *in vivo *part, gathered and processed the data, and drafted the manuscript. EB assisted in the experimental procedure and physical examination during the *in vivo *part of the experiment, was responsible for the day-to-day administration of the *in vivo *part of the study, and performed the laboratory assays and immunoassays. IK performed the histopathology and analysis. BA'tH assisted in the design of the study and helped to draft the manuscript. All authors have read and approved the final manuscript.
